# Commentary on the current status of nuclear medicine medical physics expert support in the UK

**DOI:** 10.1097/MNM.0000000000001843

**Published:** 2024-05-07

**Authors:** James W. Scuffham, John C. Dickson, Anthony Murray, Glenn D. Flux

**Affiliations:** a Department of Medical Physics, Royal Surrey NHS Foundation Trust, Guildford; b Institute of Nuclear Medicine, University College London Hospitals NHS Foundation Trust, London; c Department of Medical Physics, Bradford Teaching Hospitals NHS Foundation Trust, Duckworth Lane, Bradford and; d Department of Physics, The Royal Marsden NHS Foundation Trust, Downs Road, Sutton, UK

## Introduction

In nuclear medicine, medical physicists play a key role together with doctors, radiographers, technologists, nurses, radiopharmacists and other support staff in delivering a safe, efficacious and efficient clinical service. The role of the nuclear medicine physicist is well-defined in many publications [1–5] and typically focuses on the following areas:

1. Optimisation of diagnostic and therapeutic techniques.
2. Patient radiation safety.
3. Equipment acceptance testing and quality assurance.
4. Investigation and recommendations following patient radiation incidents.
5. Safety and radiation dosimetry of radionuclide therapies.

As with many staff groups in healthcare, there is a hierarchy within the medical physics profession. In the UK, trainee physicists commonly enter the profession via the NHS Scientific Training Programme or via an equivalence route. Upon completion of accredited training and achieving a master’s degree, medical physicists are eligible to apply for Registration with the Health and Care Professions Council (HCPC) as Clinical Scientists. However, in addition to this basic grade in medical physics, there is the legally defined role of the medical physics expert (MPE). These individuals have enhanced skills and responsibilities typically formed following several years of experience post-registration. The requirement for employers to have input from MPEs was given in the original Ionising Radiation (Medical Exposure) Regulations (IR(ME)R) 2000, although their responsibilities were sparsely defined, with guidance suggesting someone with several years’ experience would be qualified for the role. However, in the revised regulations IR(ME)R 2017, the definition of the MPE was expanded and clarified with clear areas of responsibility. Furthermore, a requirement for these individuals to be on a statutory register was introduced, with registrants obliged to submit and be assessed on a portfolio of evidence according to a well-defined list of competencies [6]. To facilitate transition, allowance was made at the time of the introduction of IR(ME)R 2017 for existing MPEs to be ‘grandfathered’ onto the register who would later be assessed, with all new applicants required to go through the portfolio and assessment process.

With responsibilities and levels of competence defined, a UK Policy Statement was published in 2022 by the Institute of Physics and Engineering in Medicine (IPEM), jointly prepared with the British Nuclear Medicine Society, British Institute of Radiology and the Administration of Radioactive Substances Advisory Committee (ARSAC). This “IPEM Policy Statement” provides guidelines on appropriate levels of MPE support specific to nuclear medicine [7]. This followed similar international guidance from the European Federation of Medical Physics (EFOMP) [8] and the International Atomic Energy Agency (IAEA) [9]. The defined levels of support in the IPEM Policy Statement are given as ranges of whole-time equivalent (WTE) staff and are dependent on the variety and complexity of work performed in a department e.g. SPECT, PET, therapy, and the size of the department primarily defined by its number of scanners. Anecdotally within the community, there is a belief that nuclear medicine departments are struggling to reach the levels of MPE support listed in the guidelines. To address this, a group of heads of nuclear medicine physics departments in the UK were asked to participate in a survey to test the hypothesis that the nuclear medicine community were not able to meet the levels of MPE expressed in guidance. This paper presents the results of this survey and provides a commentary on the current and potential future course of MPE provision in the UK.

## Method

Nuclear medicine departments in the UK were surveyed to determine their current and future expected levels of MPE support for their respective departments. Questions were asked about current levels of MPE and non-MPE medical physics staff, junior trainees supported (to understand future provision), and a self-assessment of how the department’s MPE staffing compares to the levels in the IPEM Policy Statement [7]. The survey was sent via email to a database of nuclear medicine departments that are members of a ‘Heads of Nuclear Medicine Physics’ group established in March 2022. The survey was open between July 2022 and February 2023. The six questions posed are listed in Table 1.

**Table 1:** MPE survey questions

1. How many MPEs do you currently employ? (number of individuals and overall WTE)? 2. How many Clinical Scientists who are not MPEs do you currently employ? (individuals and WTE) 3. How many non-clinical scientist medical physics staff (e.g. technologists) do you currently employ (individuals and WTE)? 4. How many Specialism STP (or equivalence) medical physics trainees are you currently supporting (individuals and WTE)? 5. How many of your medical physicists are expected to submit their MPE portfolios of evidence within the next 6 months? 6. What are the minimum and maximum levels of MPEs do you believe you need to cover all the services you support according to the ranges in the recent guidance? Please include your own site and any other sites to which you provide MPE support.

MPE, medical physics expert; STP, scientific training program; WTE, whole-time equivalent.

## Results

**Fig. 1:**
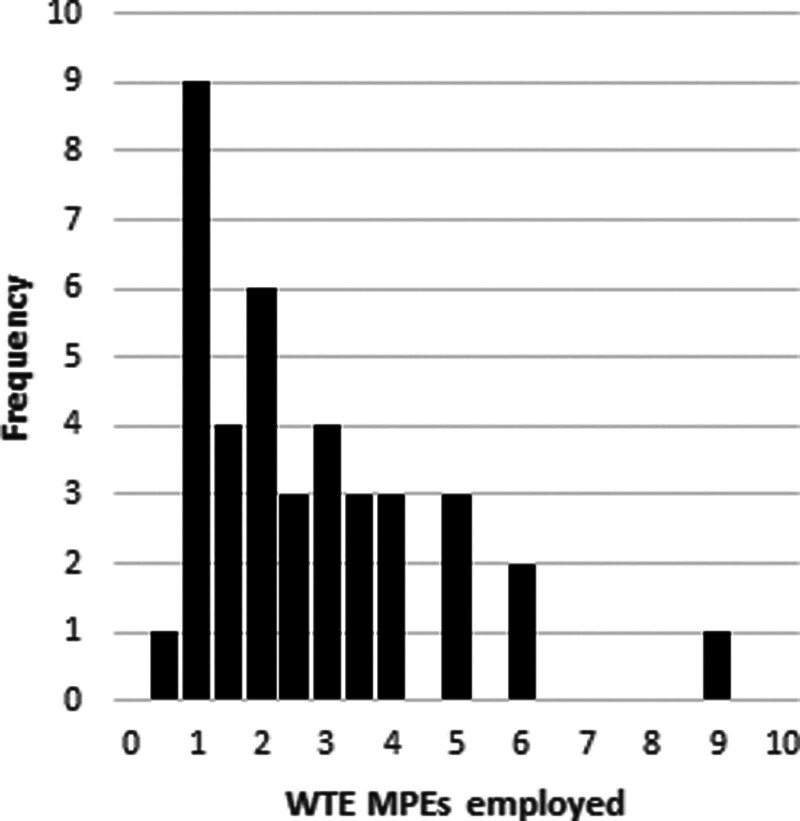
Histogram showing the number of WTE MPEs employed by respondents.

Of the 56 departments surveyed 40 responded, giving a response rate of 71%. Figure 1 shows the number of MPEs employed within surveyed departments. It was most common for departments to employ 1.0 WTE MPE with an average of 2.6 WTE (range 0.5–9.0 WTE).

**Fig. 2:**
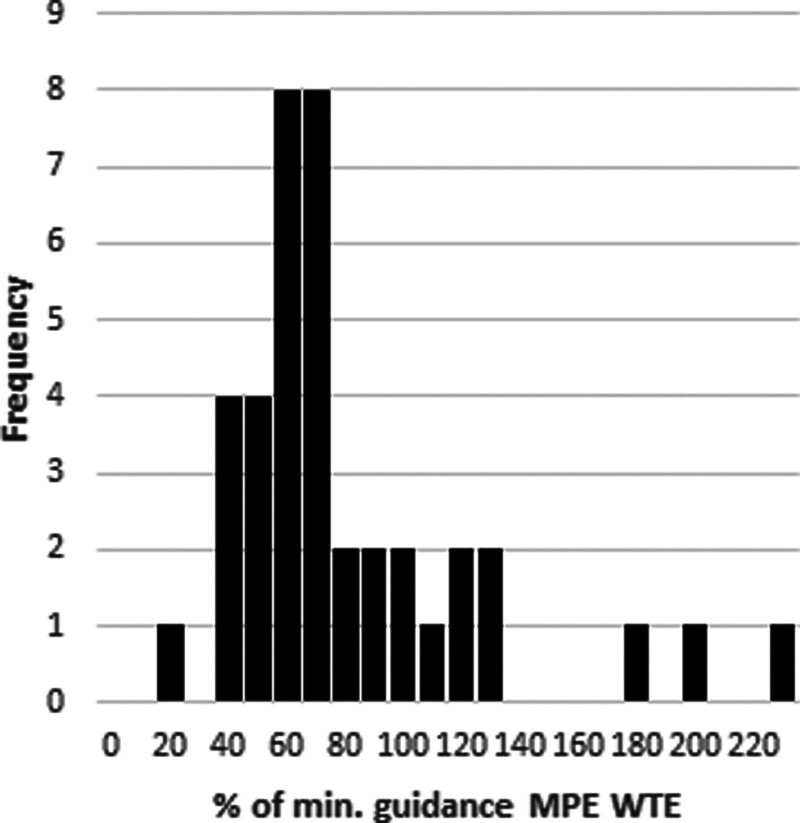
Histogram showing respondents’ current MPE staffing WTE expressed as a percentage of the minimum guidance level for their service (based on self-assessment).

Most centres did not, according to their own self-assessment, meet the minimum number of MPE as stated in IPEM Policy Statement (Fig. 2). The average level of MPE support was 75% of the minimum levels specified in the IPEM Policy Statement, with a minimum of 20% and a maximum of 220%.

**Fig. 3:**
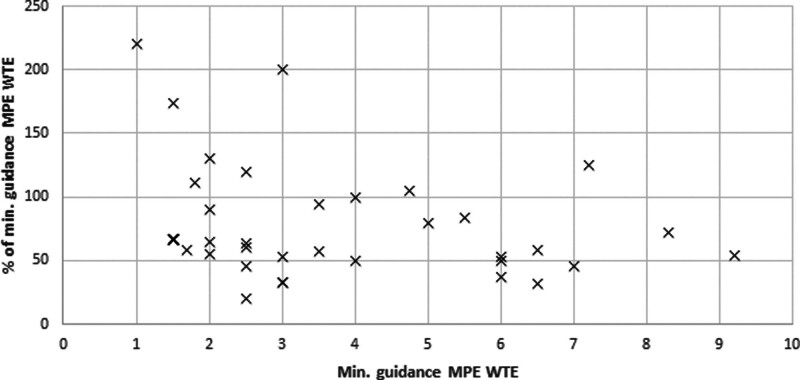
Correlation between the minimum level of MPE staffing according to departments’ self-assessment and the actual MPEs employed as a percentage of the minimum guideline level.

There was no significant correlation found between the size of the department and the percentage of minimum guideline MPEs, although smaller centres were more likely to meet the recommendations (Fig. 3).

**Fig. 4:**
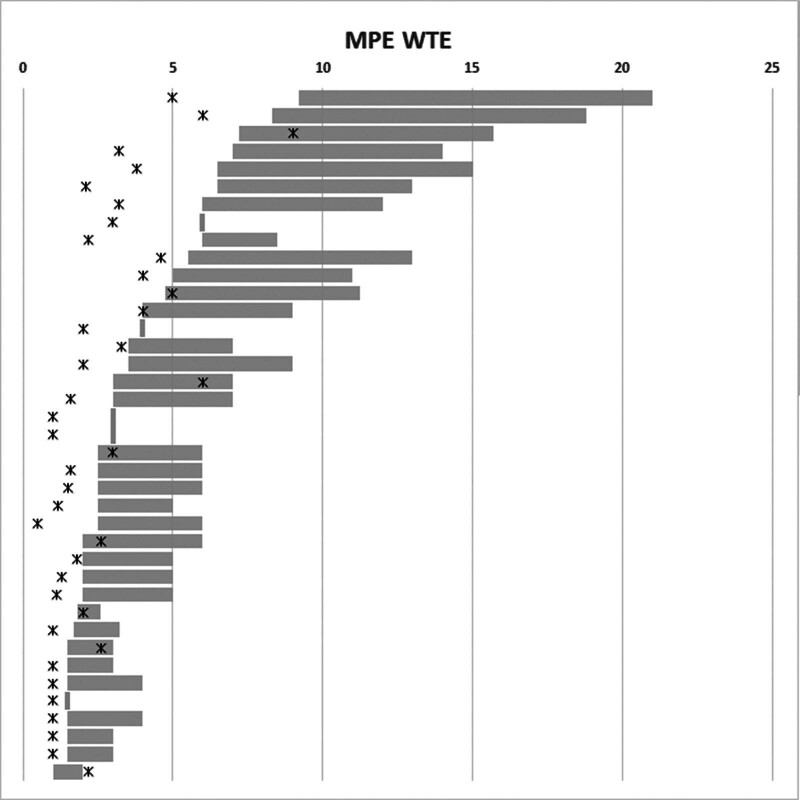
Current WTE MPEs employed (crosses) and self-assessed ranges of WTE required according to guidance (grey bars) for all respondents, ordered by minimum guidance WTE.

Eight sites (21% of respondents) had MPE numbers above the minimum recommendation (Fig. 4). It is important to note that the guidance only applies to MPEs and not Clinical Scientists, but if all HCPC-registered Clinical Scientists (not just MPEs) are included in the WTE employed, 26 sites (65%) would meet the minimum recommended WTE level, and if all medical physics staff in the department are included 31 sites (78%) of respondents meet this level.

The survey showed that an additional 45 WTE MPE would be needed to meet the self-assessed minimum recommended levels stated by survey respondents. It was also found that there were 38 trainees with specialism in nuclear medicine or imaging with ionising radiation, and that 24 physicists were expected to submit their portfolio of evidence for recognition as a MPE within the next 6 months.

## Discussion

The survey described in this paper provides a good snapshot of the current status of MPE provision in the UK, with 71% of surveyed sites responding. It is not known what percentage of all departments in the UK were captured in the survey, but the survey was widely distributed amongst the nuclear medicine physics community representing a range of different departments (including large regional centres supporting smaller departments) across all nations of the UK. We therefore believe that there is no inherent bias in the results presented.

The survey showed that the majority (79%) of sites in the UK do not meet the minimum number of MPEs defined in IPEM Policy Statement. On average sites had 75% of the minimum recommended levels although there was a wide range of between 20% and 220%. These levels of MPE support were self-declared and were not validated or checked for consistency across sites, but this is nevertheless concerning. More positive was the fact that smaller sites seemed more likely to have an appropriate level of MPE support, although it is most likely that this will be a single individual employed at 1.0 WTE, implying there may be little resilience to cover sickness and annual leave. Many smaller sites will be supported by larger regional centres with potentially more depth of staffing for resilience. Our survey asked medical physics departments to include any smaller sites that they support in their calculation of overall MPE requirements. Therefore, the data do not separate out these smaller departments that do not employ their own medical physicists. However, this approach did ensure that the overall MPE requirements for regional services were captured.

Based on supplied data it was calculated that a further 45 WTE MPE would be required to meet minimum recommendations. The number of current trainees specialising nuclear medicine was stated as 38, with 24 individuals aiming to submit their portfolio of evidence for MPE certification within the next 6 months. This will help to address the shortfall but does not account for attrition of the workforce through retirement or leaving the profession. These absolute numbers of forthcoming MPEs are not adjusted for the survey response, but we can forecast with reasonable certainty that there will be a deficit in the ‘talent pool’ of MPEs in the coming years, which will make it difficult to address the current shortfall. The process of collecting and submitting evidence for MPE portfolios takes some time, and given widespread staff shortages, this is often challenging to achieve within normal working hours. Furthermore, the process of assessment of submitted portfolios is time-consuming and performed centrally on a voluntary basis, with portfolios taking up to 6 months to assess. Streamlining the process of MPE certification should be considered, particularly given that there are individuals on the original grandfathered list who have yet to be formally assessed, and also that there is expected to be a method of re-registration based on Continuous Professional Development [10].

The IPEM Policy Statement on MPE staffing levels was compiled taking into account data on WTE MPE support levels from ARSAC Employer License applications [7]. However, quantification of WTE available MPE support is often open to interpretation. For example, a remote site might secure contracted MPE services from a larger centre and the MPE may attend the site 1 day each week, and are otherwise available for remote support. An applicant for an Employer License for such a site may enter either 0.2 or 1.0 WTE, depending on interpretation. Furthermore, the larger centre may also count the MPE that supports the remote site in their own allocation, effectively leading to double-counting and skewing of the apparent MPE coverage. The situation may be further complicated if MPEs also act as Radiation Protection Adviser, Radioactive Waste Adviser to one or multiple sites, as it is difficult to quantify the time allocated to each role. Recent revisions to the ARSAC Employer License application process have expanded the level of detail on remote- and on-site MPE support, but a risk of double-counting across centres still exists. Extrapolations based on historical ARSAC applications, without accounting for extant hub-and-spoke support structures, must therefore be treated with caution. Consideration should perhaps be given instead to adopting a more tailored, ‘bottom up’ approach, such as the methods proposed by EFOMP and the IAEA [8,9]. This may be more appropriate, particularly given that there is considerable variability in staffing mix, department structure, and the roles of physicists nationwide.

The IPEM Policy Statement [7] is now being adopted by ARSAC as the standard when evaluating MPE support levels for new applications and renewals of Employer Licenses. The authors are aware of multiple sites that have been issued with short-term licenses contingent on a review and increase of MPE support provision. However, our survey indicates that the medical physics workforce does not currently exist to fulfil these demands.

It should be welcomed that the role of the MPE and their areas of responsibility are better defined in our current legislation and that their highly valuable contribution to a safe and efficient clinical service is recognised in national guidance. However, there remains some lack of clarity around the extent to which MPEs could (or should) act in a senior supervisory role, potentially supported by a team of HCPC-registered Clinical Scientists and technologists. To draw an analogy, nuclear medicine departments are only required to entitle a single ARSAC Licensed Practitioner regardless of the size and complexity of their service. This Practitioner takes overall clinical responsibility for the medical exposures in the department, but other doctors may be entitled to carry out tasks as Operators, following protocols and guidance that have been approved by the ARSAC Licensed Practitioner. However, under current guidance, larger and more complex services are required to employ multiple MPEs, even if there is an establishment of Clinical Scientists and technologists to support their work. While both ARSAC Licensed Practitioners and MPEs hold significant responsibilities, it is clear that the responsibilities of the Practitioner is the greater of the two. One might argue that departments should also be compelled to employ more ARSAC Licensed Practitioners, but it is clear that there is currently an inconsistency in the required staffing levels across the two professional groups, yet there are significant recruitment challenges in both.

## Conclusion

Our survey has shown there is currently significant nationwide challenges in meeting current guidance on MPE staffing levels in nuclear medicine departments. Although we identified many Clinical Scientists who are close to achieving MPE certification, these are not likely to be in sufficient numbers to address the current shortfall. Streamlining the application process for MPE certification should be a priority, to ensure that staff with adequate experience can be entitled as MPEs promptly by employers. Consideration should also be given to adopting more nuanced approaches to defining acceptable levels of scientific support for nuclear medicine services that takes account not only of service size and complexity, but also of the skill mix within the scientific workforce, and hub-and-spoke support models. This is particularly important if licensing decisions are to be made on the basis of these evaluations.

## Acknowledgements

The authors wish to thank all the members of the Heads of Nuclear Medicine Physics group who contributed to this survey.

Data presented previously at the British Nuclear Medicine Society Annual Meeting 2023 and published as abstract in Nuclear Medicine Communications **44**(6):518–561, June 2023.

### Conflicts of interest

There are no conflicts of interest.
